# Sishen Wan^®^ Ameliorated Trinitrobenzene-Sulfonic-Acid-Induced Chronic Colitis *via* NEMO/NLK Signaling Pathway

**DOI:** 10.3389/fphar.2019.00170

**Published:** 2019-03-06

**Authors:** Hai-Yan Wang, Hai-Mei Zhao, Yao Wang, Yi Liu, Xiu-Yun Lu, Xue-Ke Liu, Fang Chen, Wei Ge, Zheng-Yun Zuo, Duan-Yong Liu

**Affiliations:** ^1^ Party and School Office, Jiangxi University of Traditional Chinese Medicine, Nanchang, China; ^2^ School of Basic Medical Sciences, Jiangxi University of Traditional Chinese Medicine, Nanchang, China; ^3^ Department of Microbiology and Immunology, School of Basic Medical Sciences, Heilongjiang University of Chinese Medicine, Harbin, China; ^4^ Department of Postgraduate, Jiangxi University of Traditional Chinese Medicine, Nanchang, China; ^5^ Science and Technology College, Jiangxi University of Traditional Chinese Medicine, Nanchang, China; ^6^ Affiliated Hospital of Jiangxi University of Traditional Chinese Medicine, Nanchang, China; ^7^ Key Laboratory of Pharmacology of Traditional Chinese Medicine in Jiangxi, Nanchang, China

**Keywords:** Sishen Wan, chronic colitis, NEMO/NLK signaling pathway, NF-κB, pharmacologic action

## Abstract

The nuclear factor (NF)-κB signaling pathway plays an important role in the initialization and development phase of inflammatory injuries, including inflammatory bowel disease (IBD). Sishen Wan (SSW) is a classic Chinese patent medicine listed in the Chinese Pharmacopoeia, which is usually used to treat chronic colitis; however, it is unclear whether SSW can treat IBD *via* the NF-κB signaling pathway. In the present study, the therapeutic effect of SSW was demonstrated by the decreased index of colonic weight, macroscopic and microscopic score, and pathological observation in chronic colitis induced by trinitrobenzene sulfonic acid. In colonic mucosa of rats with chronic colitis, SSW reduced the levels of calprotectin and eliminated oxidative lesions; downregulated expression of interferon-γ, interleukin (IL)-1β and IL-17; increased expression of IL-4; and suppressed expression of NF-κB p65, and NF-κB essential modulator (NEMO)-like kinase (NLK). Furthermore, SSW inhibited ubiquitinated NEMO, ubiquitin-activated enzyme, and E2i activation, and phosphorylation of downstream proteins (cylindromatosis protein, transforming growth factor-β-activated kinase and P38). These results show that the therapeutic effects of SSW in chronic colitis were mediated by inhibiting the NEMO/NLK signaling pathway to suppress NF-κB activation.

## Introduction

Sishen Wan (SSW) is used to treat chronic colitis, including inflammatory bowel disease (IBD), allergic colitis, and irritable bowel syndrome (IBS). The main characteristics of these diseases are recurrent diarrhea, hematochezia, and celialgia (Wang et al., 2008; Xie and Li, 2010). In China, SSW is a classic prescription in traditional Chinese medicine (TCM) for treatment of colitis and has been used for several thousand years. Some clinical studies have shown that SSW reached a total effective rate of 75.98% in 204 patients with IBD, and the recurrence rate was only 8.1% within 6 months, whereas the recurrence rate with salazosulfapyridine was 23.3% (Lu et al., 2011). In our previous studies, we have indicated that, in IBD, SSW exerts antioxidant and antiapoptotic activity *via* the P38 mitogen-activated protein kinase (MAPK) signaling pathway, and regulating the equilibrium between proinflammatory and anti-inflammatory cytokine networks (Liu et al., 2012, 2015a,b; Zhao et al., 2013). Nevertheless, the mechanism of action of SSW in treatment of IBD remains mostly unclear.

IBD is a frequently occurring disease in western countries. In the past 20 years, the incidence of IBD in China has markedly increased and attracted widespread attention. The incidence is 3.14/10^5^ in southern China and 1.64/10^5^ in the north (Zeng et al., 2013; Yang et al., 2014). IBD is a chronic and nonspecific disease that includes ulcerative colitis (UC) and Crohn’s disease (CD), with unknown pathogenesis. Possible causes have been reported and include genetic factors, unbalanced intestinal flora, and abnormal diet. IBD is an immunologically mediated disorder of unknown origin (Ebert et al., 2009). As a central regulator of chronic inflammation, nuclear factor (NF)-κB and its family play significant roles in the pathological process of IBD (Schreiber et al., 1998). The NF-κB family is an important therapeutic target for IBD and anti-inflammatory agents exert some of their effects by inhibiting activation of the NF-κB family. Activation of NF-κB regulates transcription of proinflammatory cytokine genes and stimulates secretion of cytokines including interferon (IFN)-γ, interleukin (IL)-1β, IL-12/23, IL-17, and tumor necrosis factor (TNF). These phenomena were induced by phosphorylation of IκB proteins by the IκB kinase (IKK) complex (IKKα, IKKβ, and NF-κB essential modulator or NEMO) (Tak and Firestein, 2001; May et al., 2002) and led to colonic mucosal damage and IBD.

Although there is little evidence to prove that SSW exerts its therapeutic effect on chronic IBD by inhibiting activation of the NF-κB signaling pathway, SSW does regulate the balance between proinflammatory and anti-inflammatory cytokines to ameliorate experimental colitis induced by trinitrobenzene sulfonic acid (TNBS). We investigated the activation of NF-κB and the NEMO/NLK (NEMO-like kinase) signaling pathway to establish the mechanism of action of SSW in IBD.

## Materials and Methods

### Animals

Male Sprague–Dawley rats weighing 180–220 g were purchased from the Animal Center of Peking University Health Science Center (animal certificate number SCXK 2006-0008). All animals were housed under pathogen-free conditions with standard laboratory chow, daily 12-h light/dark cycle, and constant room temperature, and freely bred with a standard diet and tap water according to the guidelines of the animal center. The present protocol (permit number: JZ2016-116) was approved by the Institutional Animal Care and Use Committee (IACUC) of Jiangxi University of Traditional Chinese Medicine. Rats were handled according to the guidelines on animal welfare of IACUC. All animals were acclimatized to the animal center conditions for 3 days before the experimental studies were performed. Forty rats were divided into two groups: 10 in the normal group and the other 30 rats had experimental colitis induced. After colitis induction, the rats were randomly distributed into three groups: untreated rats with TNBS-induced colitis; TNBS-induced colitis treated with SSW (TNBS + SSW); and TNBS-induced colitis treated with mesalazine (TNBS + Mes).

### Drugs

SSW (batch number 17080051) was purchased from Tongrentang Natural Medicine Co. Ltd. (Beijing, China). In 2018, Zhang et al. (2018) had finished the determination of nine major bioactive components and the quality control of SSW (batch number 17080051) by high-performance liquid chromatography coupled with electrospray tandem mass spectrometry (HPLC-ESI-MS/MS) method. And they found the contents of the nine major bioactive components (deoxyschizandrin, γ-schizandrin, schizandrin, schizandrol B, schisantherin A, psoralen, isopsoralen, evodiamine, and rutaecarpine) were 72.6, 131.5, 258.0, 71.2, 25.1, 1310.8, 1293.7, 22.2, and 24 μg/g, respectively. TNBS (batch number p2297) was obtained from Sigma (St. Louis, MO, USA), and mesalazine (batch number: H19980148) from Sunflower Pharma (Jiamusi, China).

### TNBS-induced Chronic Colitis

Chronic colitis was induced by TNBS as described previously (Segain et al., 2003; Gambero et al., 2007; Thomaz et al., 2009). On day 1, the rats were anesthetized by pentobarbital sodium (40 mg/kg), and colitis was induced by intracolonic instillation of TNBS-ethanol solution (3 mg TNBS dissolved in 0.3 ml 50% ethanol). TNBS-ethanol solution was injected into the colon ~8 cm from the anus. The rats were kept in a head-down position for 10 min. On days 14 and 28, the above procedures were performed again. Control animals in the normal group were treated with the same volume of 0.3 ml 50% ethanol.

### Therapeutic Protocols

According to dose selection of SSW in previous study (Liu et al., 2012), 5 g/kg dose of SSW is effective and stable to effectively treat experimental colitis. In the present study, 24 hours after the last instillation of TNBS, the rats in the TNBS + SSW group were administrated 5 g/kg SSW suspension for 10 days, which was dissolved in physiological saline. The rats in the TNBS + Mes group were treated with 300 mg/kg mesalazine for 10 days. Rats in the Normal and TNBS groups received the same volume of physiological saline by gavage for 10 days. On day 40, all rats were killed after anesthesia with pentobarbital sodium.

### Macroscopic Evaluation

The colons were removed rapidly from the rats after death, measured, and opened longitudinally along the colonic mesentery to clear any excrement. The colonic weight index (*n* = 8) was calculated according to colonic weight/body weight × 100%. The macroscopic evaluation was performed as described previously (Butzner et al., 1996). The scores for macroscopic colonic damage were as follows: 0, normal appearance; 1, focal hyperemia, no ulcers; 2, ulceration without hyperemia or bowel wall thickening; 3, ulceration with inflammation at one site; 4, ≥2 sites of ulceration and inflammation; 5, major sites of damage extending >1 cm along the length of the colon; and 6–10) damage extended to >2 cm along the length of the colon, increasing the score by 1 for each additional 1 cm of damage.

### Hematoxylin and Eosin (H&E) Staining and Microscopic Evaluation

The colonic segments were preserved and fixed in 4% polyformaldehyde solution for 7 days, and then dehydrated and embedded in paraffin. Blocks were serially sectioned at 5 μm along the cephalocaudal axis of the gut to the level of the lumen. The 5-μm section was stained with H&E. The pathological features of the colon were observed and evaluated under an Olympus microscope. A histological injury score (*n* = 8) was developed according to the standards reported by Schmidt et al. (2010). The total score included inflammatory cell infiltration and tissue damage. The scores for inflammatory cell infiltration were: 0, no infiltration; 1 increased number of inflammatory cells in the lamina propria; 2, inflammatory cells extending into the submucosa; and 3, transmural inflammatory cell infiltration. The scores for colonic tissue damage were: 0, no mucosal damage; 1, discrete epithelial lesions; 2, erosions or focal ulcerations; and 3, severe mucosal damage with extensive ulceration extending into the bowel wall.

### Preparation of Colonic Tissue Supernatant

The segmental colonic tissues (*n* = 8) were lysed in RIPA buffer with protease and phosphate inhibitor cocktail (eBioscience, San Diego, CA, USA), and homogenated by ultrasound. Subsequently, the homogenate was centrifuged at 30,000 g for 40 min at 4°C, and colonic supernatant was extracted. The supernatants were used to analyze antioxidant level, cytokine secretion, and target protein expression.

### Antioxidant Analysis

The colonic supernatants were used for antioxidant analysis by coomassie brilliant blue. Superoxide dismutase (SOD), glutathione peroxidase (GSH-PX), total antioxidant capacity (T-AOC), myeloperoxidase (MPO), malondialdehyde (MDA), NO, and induced NO synthase (iNOS) were detected by ultraviolet spectroscopy. Endogenous NO synthase (eNOS) was measured by ELISA.

### ELISA

The other supernatant (*n* = 8) was used to detect the level of IFN-γ, IL-1β, IL-4, IL-17, and calprotectin by ELSIA (R&D Systems, Minneapolis, MN, USA). Absorbance at 450 nm was read by microplate reader (Bio-Rad, Hemel Hempstead, UK). Each supernatant was analyzed in duplicate.

### Western Blotting

Colonic biopsy specimens were homogenized and supernatant was extracted as described above. Protein concentration (*n* = 6) was determined by classic BCA protein assay (Beyotime, Nanjing China). Equal amounts of total protein (60–80 μg) were subjected to SDS-PAGE and transferred to polyvinylidene fluoride membranes using Bio-Rad western blotting apparatus. After blocking with 5% fat-free milk or bovine serum albumin, the membranes were probed with the following primary antibodies overnight at 4°C. The antibodies were anti-GAPDH (1:3000), anti-P65 (1:2000), anti-NLK (1:2000), ubiquitinated NEMO (IKKγ) (1:1000), ubiquitinated NLK (1:1500), anti-E1 (ubiquitin-activated enzyme), (1:1500), anti-E2i (1:1500), anti-E2D (1:1500), anti-E3 (ubiquitin protein ligase) (1:1500), anti-TAK (transforming growth factor-β activated kinase) (1:2000), anti-phospho (p)-TAK (1:2000), anti-STAT3 (signal transducer and activator of transcription 3) (1:2000), anti-p-STAT3 (1:2000), anti-P38 (1:1000), and anti-p-P38 (1:1000). All primary antibodies were purchased from Abcam. This was followed by incubation with the secondary antibody (1:2000–1:4000) (Abcam) for 1 h at 37°C. The labeled protein bands were scanned with the HP Scanjet 5,500 (Hewlett Packard France, Les Ullis, France). Finally, the relative protein concentration was analyzed by the gray level of the band by Quantity One version 4.40 software (Bio-Rad).

### Statistical Analysis

All parameters were expressed as mean ± SEM. All statistical analyses were performed using GraphPad Prism software. Significance was determined using one-way ANOVA followed by the Tukey test for multiple comparisons. *p* < 0.05 was considered to be statistically significant, although in some cases higher levels of significance were noted and described in the figure legends where applicable: *p < 0.05, **p < 0.01 versus Normal, and ^#^ *p* < 0.05, ^##^ *p* < 0.01 versus TNBS.

## Results

### SSW Ameliorated TNBS-induced Chronic Colitis

**Figure 1:**
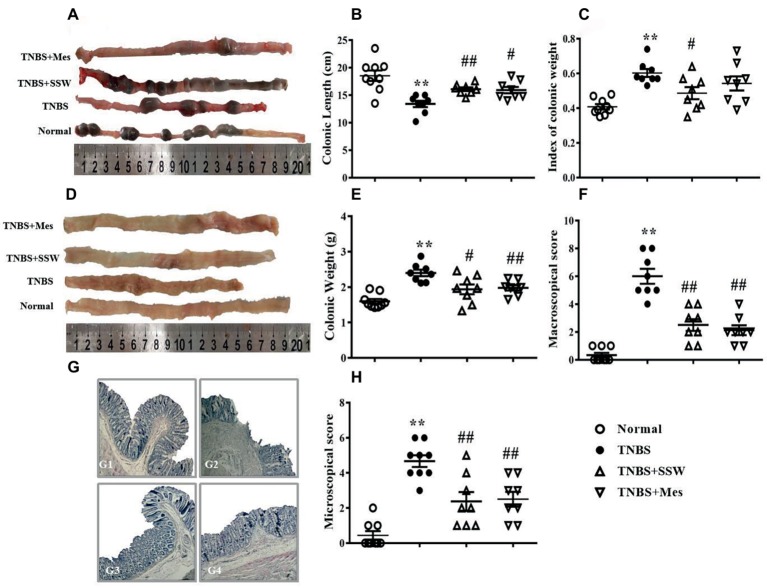
Therapeutic evaluation of SSW-treated chronic colitis in rats. **(A)** Typical images of intact colon. **(B)** Colonic length. **(C)** Index of colonic weight. **(D)** Macroscopic view of the opened colon. **(E)** Colonic weight. **(F)** Macroscopic score. **(G)** Typical histological images stained by H&E, bar = 100 μm. G1: the histological image of colonic tissue of a rat in the Normal group; G2: the histological image of colonic tissue of a rat in the TNBS group; G3: the histological image of colonic tissue of a rat in the TNBS+SSW group; G4: the histological image of colonic tissue of a rat in the TNBS+Mes group. **(H)** Microscopic score. Data are presented as mean ± SEM (*n* = 8). *p < 0.05 and **p < 0.01 versus the Normal group; ^#^ *p* < 0.05 and ^##^ *p* < 0.01 versus the TNBS group.

TNBS-induced chronic colitis is a classic animal model with recurrent characteristics including diarrhea, hematochezia, weight loss, and hair loss. These characteristics were seen in the present study. Colonic length was shorter (Figures 1A,B) and colonic weight (Figure 1E) and index of colonic weight (Figure 1C) were increased in rats in the TNBS groups compared with the Normal group. When the TNBS + SSW and TNBS + Mes groups were compared, colonic length was restored (Figures 1A,B), and colonic weight (Figure 1C) and index of colonic weight (Figure 1E) were decreased. When we cut the colon along the mesentery, we found multiple ulcer formation, thickened bowel wall, hyperemia, and edema in the mucosa from rats in the TNBS groups (Figure 1D). Pathological observation found classic IBD characteristics as follows: disordered arrangement of the colonic gland, anabrotic or deciduous epithelial tissue, plentiful inflammatory cell infiltration, hemangiectasis, and fibrous hyperplasia (Figure 1G), although the extent of pathological damage was alleviated (Figures 1D,G). Importantly, compared with rats in the TNBS group, the scores for macroscopic and microscopic evaluation (Figures 1D,F–H) were markedly reduced in the TNBS + SSW and TNBS + Mes groups. The results suggested that SSW relieved the pathological damage in the colon in rats with TNBS-induced chronic colitis.

### SSW Protected against Oxidative Damage in Colonic Tissue Induced by TNBS

**Figure 2:**
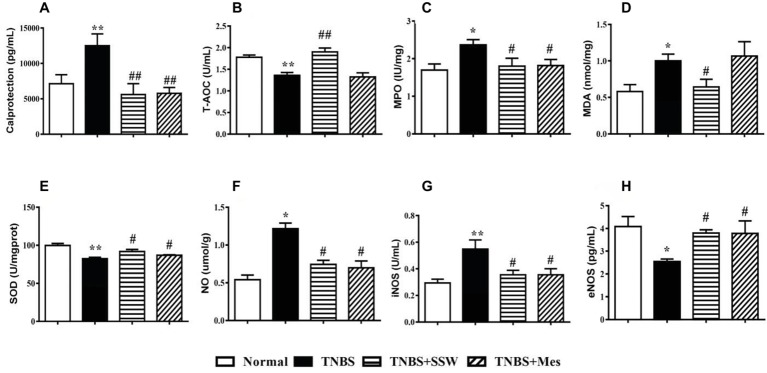
Level of calprotectin and antioxidants. **(A)** Calprotectin; **(B)** T-AOC; **(C)** MPO; **(D)** MDA; **(E)** SOD; **(F)** NO; **(G)** iNOS; **(H)** eNOS. Data were presented as mean ± SEM (*n* = 8). *p < 0.05 and **p < 0.01 versus the Normal group; ^#^ *p* < 0.05 and ^##^ *p* < 0.01 versus the TNBS group.

The levels of calprotectin and oxidative stress in colonic mucosa are two important parameters for evaluating the extent of colonic tissue injury. Ten days after the third intracolonic instillation of TNBS, the levels of calprotectin in colonic mucosa from rats with untreated colitis were increased when they were compared with those in rats with colitis treated by SSW (Figure 2A). Similar changes were seen for the activity of MPO (Figure 2C), MDA (Figure 2D), NO (Figure 2F), and iNOS (Figure 2G). The levels of T-AOC (Figure 2B), SOD (Figure 2E), and eNOS (Figure 2H) in colonic mucosa of untreated rats with colitis were lower than those in the TNBS+SSW and TNBS+Mes groups. Oxidative damage of the colon was successfully induced by TNBS, and alleviated by administration of SSW and Mes.

### SSW Regulated Secretion of Cytokines in Colonic Tissues

**Figure 3:**
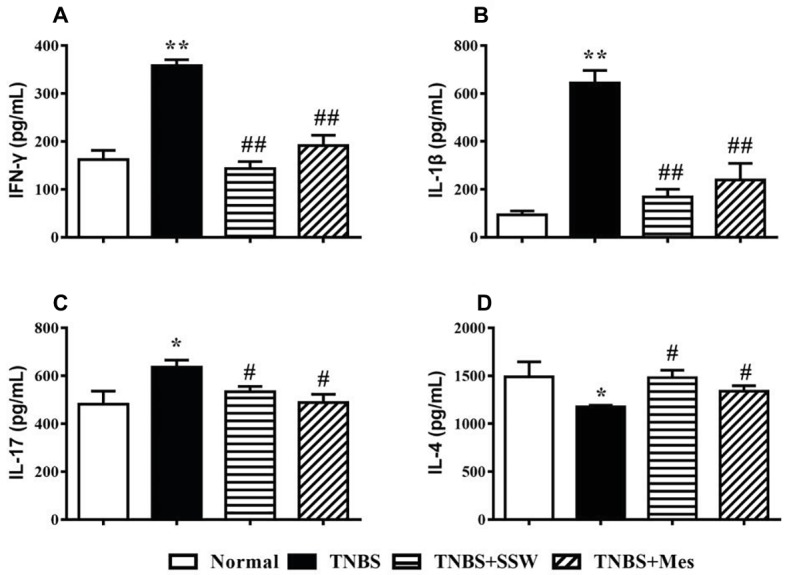
Expression of IFN-γ, IL-1β, IL-17, and IL-4. **(A)** IFN-γ; **(B)** IL-1β; **(C)** IL-17; **(D)** IL-4. Data were presented as mean ± SEM (*n* = 8). *p < 0.05 and **p < 0.01 versus the Normal group; ^#^ *p* < 0.05 and ^##^ *p* < 0.01 versus the TNBS group.

Imbalance of cytokines in inflammatory injury of colonic mucosa is a significant feature in the pathogenesis of IBD. Expression of IFN-γ (Figure 3A), IL-1β (Figure 3B), and IL-17 (Figure 3C) in the colon from rats in the TNBS group was higher than in the Normal group. Expression of IL-4 was markedly decreased in colonic tissues from rats with untreated colitis (Figure 3D). In rats with colitis rats treated with SSW, expression of IFN-γ (Figure 3A), IL-1β (Figure 3B), and IL-17 (Figure 3C) was suppressed, and expression of IL-4 (Figure 3D) was increased. These results suggested that SSW regulated the balance of cytokines in colonic mucosa from rats with TNBS-induced colitis.

### SSW Inhibited Ubiquitination of NEMO/NLK Signaling Pathway in Colonic Tissues

**Figure 4:**
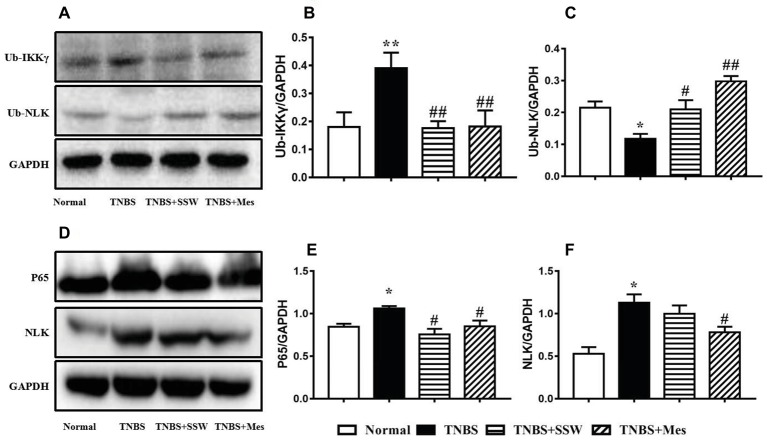
Western blot analysis of ubiquitinated (Ub)-IKKγ (NEMO), Ub-NLK, NF-κB P65, and NLK. **(A)** Western blotting of Ub-IKKγ and Ub-NLK. **(B)** Quantitative analysis of Ub-IKKγ. **(C)** Quantitative analysis of Ub-NLK. **(D)** Western blotting of NF-κB P65 and NLK. **(E)** Quantitative analysis of NF-κB P65. **(F)** Quantitative analysis of NLK. Data were presented as mean ± SEM (*n* = 6). *p < 0.05 and **p < 0.01 versus the Normal group; ^#^ *p* < 0.05 and ^##^ *p* < 0.01 versus the TNBS group.

As a major member of the IKK family, ubiquitination of the NEMO (IKKγ)/NLK signaling pathway promoted activation of NF-κB in the inflammatory response to IBD. As shown in the Figure 4, after rats were treated with TNBS three times, NEMO protein was ubiquitinated and highly expressed (Figures 4A,B), while NLK ubiquitination was inhibited in the damaged colonic mucosa of rats with untreated colitis (Figures 4A,C). High-level NEMO ubiquitination limited ubiquitinated NLK expression and activated NF-κB, and expression of NF-κB P65 (Figures 4D,E) and NLK (Figures 4D,F) was increased when compared with normal control rats. Treatment with SSW and Mes inhibited ubiquitination of NEMO and increased expression of NLK in colonic tissue. SSW inhibited NF-κB P65 activation in colonic mucosa in rats with colitis. However, the level of NLK protein was not obviously changed after treatment of colitis with SSW. These results showed that SSW inhibited ubiquitination of the NEMO/NLK signaling pathway to deactivate NF-κB in colonic mucosa from rats with TNBS-induced colitis.

### SSW Regulated Ubiquitin-Proteasome System Activation in Colonic Tissues

**Figure 5:**
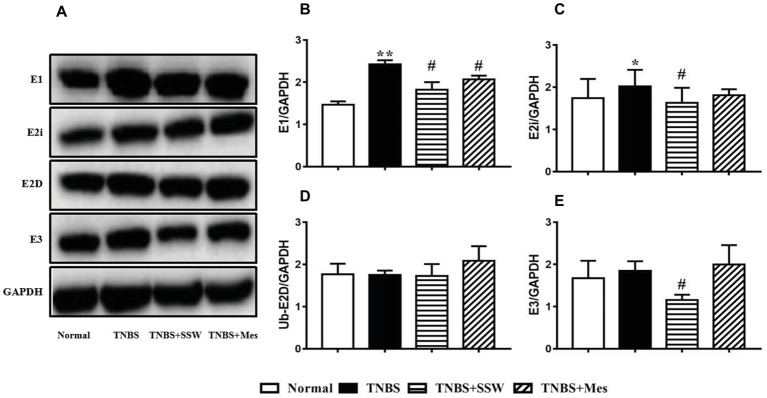
Western blot analysis of E1, E2i, E2D, and E3. **(A)** Western blotting of E1, E2i, E2D, and E3. **(B)** Quantitative analysis of E1. **(C)** Quantitative analysis of E2i. **(D)** Quantitative analysis of E2D. **(E)** Quantitative analysis of E3. Data were presented as mean ± SEM (*n* = 6). *p < 0.05 and **p < 0.01 versus the Normal group; ^#^ *p* < 0.05 versus the TNBS group.

The ubiquitin-proteasome system promotes ubiquitin molecules to connect with target protein, and includes ubiquitin, E1, E2, and E3. During the development of TNBS-induced colitis, E1 (Figures 5A,B) and E2i (Figures 5A,C) were activated and upregulated, which contrasted with the normal control rats. Compared with rats in the TNBS groups, SSW inhibited expression of E1 (Figures 5A,B), E2i (Figures 5A,C), and E3 (Figures 5A,E) in colonic tissue of rats with experimental colitis. The results indicated that SSW controlled the ubiquitin-proteasome system activation to limit ubiquitination of the NEMO/NLK signaling pathway in TNBS-induced colitis.

### Effect of SSW on Downstream Proteins of NEMO/NLK Signaling Pathway in Colonic Tissues

The NEMO/NLK signaling pathway was activated to induce a chain reaction of its downstream proteins, especially activation of TAK, STAT3, and P38. The levels of cylindromatosis protein (CYLD) (Figures 6A,B), p-TAK (Figures 6A,C) and p-P38 (Figures 6A,E), and the ratios of p-TAK/TAK (Figure 6D) and p-P38/P38 (Figure 6F) in the colonic mucosa of rats with untreated colitis were higher than those in the normal control rats. However, after treatment with SSW and Mes, phosphorylation of downstream proteins (TAK, CYLD, and P38) (Figures 6A–C,E) was inhibited, and the ratios of p-TAK/TAK and p-P38/P38 (Figures 6D,F) were downregulated compared with those in the TNBS group. The results showed that SSW inhibited activation of downstream proteins of the NEMO/NLK signaling pathway in TNBS-induced chronic colitis.

**Figure 6:**
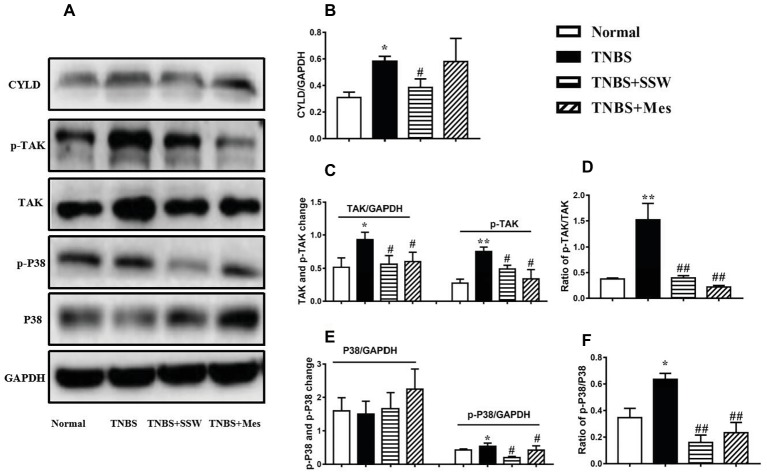
Western blot analysis of CYLD, p-TAK, TAK, p-P38, and P38. **(A)** Western blotting of CYLD, p-TAK, TAK, p-P38, and P38. **(B)** Quantitative analysis of CYLD. **(C)** Quantitative analysis of p-TAK and TAK. **(D)** Ratio of p-TAK/TAK. **(E)** Quantitative analysis of p-P38 and P38. **(F)** Ratio of p-P38/P38. Data were presented as mean ± SEM (*n* = 6). *p < 0.05 and **p < 0.01 versus the Normal group; ^#^ *p* < 0.05 versus the TNBS group.

## Discussion

In the present study, experimental colitis induced by TNBS showed the characteristic pathological features of chronic colitis in the colonic mucosa, including ulceration, hyperplasia, and inflammatory cell infiltration. In the process of chronic inflammation, high expression of NF-κB P65, imbalance of cytokines, and oxidative damage were simultaneously found, which suggested that activation of NF-κB P65 contributed to excess secretion of cytokines, and induced inflammatory damage with over oxidation to form colonic pathological injury. These findings are consistent with previous studies (Murano et al., 2000).

In TNBS-induced colitis, the colonic mucosa had markedly increased expression of ubiquitinated IKKγ, E1, E2, and E3 compared with normal colonic mucosa, while ubiquitinated NLK level was downregulated. These results show that overactivated ubiquitination of the NEMO/NLK signaling pathway plays a crucial role in the pathogenesis of TNBS-induced colitis.

NEMO is one of the IKK family proteins (also named IKKγ); it is a noncatalytic regulatory submit, (Rothwarf and Karin, 1999; May et al., 2002;) that is essential for activation of the NF-κB signaling cascade. NEMO has multiple protein domains and forms dimers, trimers, and tetramers, which can integrate various signals and bind to adaptor molecules or kinases (IKKα/β). Apart from various oxidative stress reactions (Bubici et al., 2006), NEMO plays a crucial role in activation of the NF-κB signaling pathway, which can cause signal-induced activity of the intact IKK complex to transduce a subset of classical NF-κB signals. In this process, under effects of special stimulus, post-translationally modified NEMO can activate the NF-κB signaling pathway by ubiquitination, phosphorylation, and sumoylation (Tegethoff et al., 2003; Vinolo et al., 2006; Fontan et al., 2007). Some studies have shown that dissociation of NEMO from the IKK complex using a cell-permeable peptide spanning the NEMO-binding domain blocks proinflammatory NF-κB activation (Jimi et al., 2004). So, NEMO is an essential modulator that activates the NF-κB signaling pathway.

Ubiquitin is a 76-amino-acid protein that binds to target proteins including E1, E2, and E3, and is eventually recognized and degraded by 26S proteasome. The above four proteins ultimately form the ubiquitin-proteasome system to regulate cell signal transduction (Chen and Sun, 2009). NEMO ubiquitination controlled activation of the NF-κB signaling pathway by positive and negative feedback of ubiquitylation and deubiquitylation. For positive control, E2 binds to E3 and specifically recognizes the phosphorylated IκB. As a representative molecule of E2, K63-ubiquitinated protein (Ub^K63^) activates TAK1 and IKK to promote high expression of BCL10 and nucleotide-binding oligomerization domain-containing protein 2, eventually leading to activation of the NF-κB signaling pathway (Yamada et al., 2006; Kanneganti et al., 2007; Sebban-Benin et al., 2007; Gautheron and Courtois, 2010). The E3 ligase linear ubiquitin chain assembly complex (LUBAC) facilitates NEMO linear ubiquitination upon genotoxic stress. Inhibition of LUBAC function interrupts the genotoxic NF-κB signaling cascade by attenuating activation of IKK and TAK1 (Tokunaga et al., 2009). For negative control, ubiquitin zinc-finger protein A20 and CYLD hydrolyze TNF receptor-associated factor (TRAF)2, TRAF6, and Ub^K63^ on NEMO to yield deubiquitination of NEMO, which results in inactivation of the NF-κB signaling pathway (Vereecke et al., 2009; Sun, 2010).

NLK is an MAPK-like kinase that belongs to the proline-directed serine/threonine protein kinase superfamily, which consists of MAPKs and cyclin-dependent kinases (Brott et al., 1998; Ishitani et al., 1999). The NEMO/NLK signaling pathway is important for regulation of cell growth, development, and death. TAK1 is a downstream protein of the NEMO signaling pathway, and participates in P38 MAPK and NLK activation to regulate the Wnt/β-catenin signaling pathway by ubiquitination of TCE and LET, or as a common central medium to mediate DNA injury induced by the NF-κB signaling pathway and P38 MAPK activation, and to initiate the cascade response of the NF-κB signaling pathway (Ohkawara et al., 2004; Keren et al., 2006; Ohnishi et al., 2010; Yang et al., 2011). The NF-κB signaling pathway plays a central role in the secretion of many cytokines, and destroys the balance of proinflammatory and anti-inflammatory cytokines. In TNBS-induced colitis, cytokine imbalance was demonstrated by high expression of proinflammatory cytokines (TNF-α, IFN-γ, IL-1β, and IL-17) and low expression of anti-inflammatory cytokines (IL-4 and IL-10) to induce inflammatory damage to the colonic mucosa.

In the present study, colonic mucosa from rats with TNBS-induced colitis had increased levels of ubiquitinated IKKγ (NEMO), NF-κB P65, and NLK and decreased level of ubiquitinated NLK. Furthermore, E1 and E2i were activated and highly expressed. In this process, downstream proteins (TAK1 and P38) were phosphorylated and activated; however, CYLD was not ubiquitinated and hydrolyzed to maintain high expression. These results suggest that the mechanisms of NEMO ubiquitin-binding domains with E1 and E2 were initiated and played an essential role in activation of the NF-κB signaling pathway in TNBS-induced colitis.

There are only a few reports of SSW regulation of the NF-κB pathway to treat various diseases (Liu et al., 2012, 2015a,b). It is reported that the main effective constituents of SSW, which include rutaecarpine, evodiamine, schisandrin B, and psoralen, have similar effects in different diseases. For example, rutaecarpine significantly decreased NF-κB protein levels in liver tissues and plasma inflammatory cytokines levels to ameliorate pathological changes in the liver, and blocked NF-κB p50/p65 in human colorectal cancer (Nie et al., 2016; Sui et al., 2016). Evodiamine inhibited IκBα phosphorylation to inactivate NF-κB expression to relieve zymosan-induced inflammation (Fan et al., 2017). Schisandrin B or psoralen ameliorated chondrocyte inflammation and osteoarthritis, oxidative stress injury, and DSS-induced colitis *via* suppression of the NF-κB/MAPK signaling pathways, followed by decreased inflammatory cytokines (Liu et al., 2015a,b; Li et al., 2017; Zhu et al., 2017; Ran et al., 2018). We have previously found that SSW inhibited P38 MAPK mRNA expression to downregulate colonic epithelial cell apoptosis in colonic mucosa in mice with colitis (Zhao et al., 2013). We deduced from the above that SSW may treat IBD by inhibiting activation of the NF-κB signaling pathway; however, the mechanism of action remains unclear.

SSW contains *Semen psoraleae*, *Fructus evodiae*, *Semen myristicae,* and *Schisandra chinensis;* it was usually used to treat chronic colitis and showed a therapeutic effect in animal models of TNBS- or DSS-induced colitis (Liu et al., 2012, 2015a,b). In the present study, expression of ubiquitinated IKKγ (NEMO), NF-κB P65, and NLK was inhibited in the colonic mucosa of rats with experimental colitis treated by SSW, while the levels of E1, E2i, and E3 were decreased, and synchronously the phosphorylation of TAK1 and P38 was limited. These results show the therapeutic effect of SSW in TNBS-induced chronic colitis. We found that SSW inhibited ubiquitination of the NEMO/NLK signaling pathway to inactivate the NF-κB pathway. SSW inhibited activation of NF-κB, which led to cytokine equilibrium and amelioration of inflammatory injury in colonic mucosa.

In summary, SSW inhibition of the NEMO/NLK signaling pathway may be an effective treatment strategy in IBD.

## Author Contributions

Co-first authors H-YW and H-MZ who are primary authors of the manuscript contributed equally to this work, and designed and performed the research. D-YL and Z-YZ conceived and designed the experiments. H-YW, YW, YL, H-MZ, YL, X-YL, X-KL, FC, and WG performed the experiments. D-YL and H-MZ contributed reagents, materials and analytical tools. D-YL and Z-YZ analyzed the data. D-YL, Z-YZ, and H-YW wrote the paper.

### Conflict of Interest Statement

The authors declare that the research was conducted in the absence of any commercial or financial relationships that could be construed as a potential conflict of interest.
